# A rapid approach to detect methicillin-resistant and susceptible *Staphylococcus aureus* directly from clinical specimens

**DOI:** 10.1128/spectrum.00487-26

**Published:** 2026-08-04

**Authors:** Shivani Sharma, Habeeb D. Salaudeen, Eric M. Ransom, J. Scott VanEpps, Siu-Tung Yau

**Affiliations:** 1 Rapidect Inc., Solon, Ohio, USA; 2 Department of Electrical Engineering and Computer Science, Cleveland State University 2564, Cleveland, Ohio, USA; 3 University Hospitals Cleveland Health, Cleveland, Ohio, USA; 4 Department of Pathology, Case Western Reserve University School of Medicine 12304, Cleveland, Ohio, USA; 5 Department of Emergency Medicine, University of Michigan 1259, Ann Arbor, Michigan, USA; 6 Weil Institute for Critical Care Research and Innovation, University of Michigan 1259, Ann Arbor, Michigan, USA; 7 Biointerfaces Institute, University of Michigan 1259, Ann Arbor, Michigan, USA; University of Mississippi Medical Center, Jackson, Mississippi, USA

**Keywords:** MRSA, MSSA, *Staphylococcus aureus*, *mecA*, PBP2a, colonization screening, nasal swabs, diagnostic testing, clinical testing, rapid testing, molecular testing, culture screening, Staph

## Abstract

Screening of methicillin-resistant/susceptible *Staphylococcus aureus* (MRSA/MSSA) is important for healthcare facilities to prevent hospital-acquired infections. The two common MRSA screening approaches are culture-based testing with selective and/or differential properties and nucleic acid amplification testing (NAAT). Both approaches have limitations. Here, we evaluate a novel culture-independent, antibody-based technology, the rapid detection-analysis platform (RDAP), to detect and differentiate MRSA and MSSA. A cohort of 64 clinical specimens was used to compare the RDAP with either culture using the chromogenic BBL CHROMagar MRSA II/CHROMagar SA bi-plate (30 specimens) or NAAT using the GeneXpert MRSA NxG (34 specimens) with a blinded design. Compared to culture, RDAP correctly detected 4/4 MRSA-positive samples, 2/2 MSSA-positive samples, and 19/24 *S. aureus*-negative specimens and had an overall agreement of 83.3% (25/30). Compared to NAAT, RDAP correctly identified 4/4 MRSA-positive samples and 28/30 MRSA-negative specimens and had an overall agreement of 94.1% (32/34). All nine discrepancies were false positives, which may be explained by RDAP having a lower limit of detection than the reference methods. The negative predictive values and test sensitivities were 100% for the RDAP compared to both reference approaches. Importantly, RDAP was able to detect and differentiate MRSA and MSSA with a turnaround time of 2.3 h, significantly shorter than the turnaround time of 24 h for culture. Taken together, RDAP can provide rapid results with a high negative predictive value to reduce unnecessary broad-spectrum antimicrobial use, minimizing the risk of resistance development, adverse effects, and costs.

**IMPORTANCE:**

Methicillin-resistant *Staphylococcus aureus* (MRSA) and methicillin-susceptible *Staphylococcus aureus* (MSSA) are commonly encountered bacterial pathogens that lead to healthcare-associated infections and life-threatening conditions. Healthcare systems often implement active patient carriage screening programs to identify MRSA, and sometimes MSSA, using nucleic acid amplification testing or culture-based diagnostic approaches. Here, the rapid detection-analysis platform (RDAP), a novel bacterial detection approach, was evaluated for detecting and distinguishing MRSA and MSSA directly from clinical carriage nasal swab specimens. The RDAP approach offers promising results significantly faster than culture-based screening methods. The RDAP is a new technology to rapidly screen MRSA and MSSA to improve infection prevention practices and potentially aid in therapeutic decision-making.

## INTRODUCTION

*Staphylococcus aureus* is an opportunistic bacterial pathogen and one of the most commonly encountered microorganisms in clinical microbiology laboratories (1). Some *S. aureus* strains can acquire the *mecA* gene that encodes for the penicillin-binding protein 2a (PBP2a), leading to resistance to most beta-lactam antibiotics (2). These strains are collectively referred to as methicillin-resistant *Staphylococcus aureus* (MRSA), as opposed to methicillin-susceptible *Staphylococcus aureus* (MSSA). *S. aureus* can also develop resistance to other classes of antibiotics (3). MRSA infections are more difficult to treat than MSSA infections, can cause sepsis or death (4), and therefore are considered a serious threat to public health (5). Distinguishing MRSA from MSSA is not only important for therapeutic decision-making but is also useful for infection prevention and control surveillance (4, 5). It is not uncommon for health systems to implement active patient screening for MRSA and/or MSSA carriage as an essential component of transmission mitigation programs.

Two common approaches used by clinical microbiology laboratories to screen for MRSA, and sometimes MSSA, are culture in selective and/or differential media and direct-from-specimen nucleic acid amplification tests (NAATs) for *mecA*. Culture-independent NAATs offer a faster turnaround time (TAT) of 2–4 h (6) at a higher reagent expense, and culture-based tests offer less expensive reagents but with a slower TAT, i.e., 18–24 h (6). There is a diagnostic need for a rapid and cost-effective approach to identify MRSA and MSSA directly from clinical specimens.

We previously developed a culture-independent rapid detection-analysis platform (RDAP) for the detection of bacterial pathogens (7, 8). We have used the RDAP to aid in the diagnosis and treatment of bloodstream infections, including antibiotic susceptibility testing on clinical blood specimens that contained a range of gram-negative and gram-positive pathogens (8). In this article, in a proof-of-concept study, we demonstrate the feasibility of using the RDAP to detect and differentiate MRSA and MSSA from 64 clinical specimens. Results were compared to two reference methods routinely performed in our clinical microbiology laboratory: BBL CHROMagar MRSA II/CHROMagar SA bi-plate (agar plating) and GeneXpert MRSA NxG, a NAAT-based technology. The study was conducted with a simultaneous blinded design, with the primary outcomes of percent agreement and TAT.

## MATERIALS AND METHODS

### Clinical specimens

This study received institutional review board approval for waiver of consent. The clinical specimens were obtained from the Pathology Biorepository via the Clinical Microbiology Laboratory at University Hospitals (UH) Cleveland Health System in 2024 and 2025. The specimens were nasal specimens for *S. aureus* and MRSA colonization screening tests, collected with Copan ESwabs, and transported to the clinical microbiology laboratory using routine clinical practices.

### Standard-of-care clinical testing

Two reference methods are used by the Clinical Microbiology Laboratory to detect MRSA and MSSA. The first is culture-based agar plating. Ten milliliters of the specimen suspension is plated on each side of the BBL CHROMagar MRSA II/CHROMagar SA bi-plate (Becton Dickinson Biosciences, San Diego, CA, USA). The bi-plate can detect and distinguish MSSA and MRSA. In our laboratory, the bi-plate is used for *S. aureus* and MRSA colonization screening, not for culturing sites of infection. The plates are incubated at 35°C for 20 h on the Kiestra Total Laboratory Automation System (Becton Dickinson Microbiology Systems). For the purposes of this study, MSSA and MRSA results were confirmed by matrix-assisted laser desorption ionization–time-of-flight mass spectrometry system (Bruker Sirus, Bruker, Billerica, MA, USA) and PBP2a lateral flow assay (Hardy Diagnostics, Santa Maria, CA, USA).

The second reference method is the NAAT-based Xpert MRSA NxG assay (Cepheid, Sunnyvale, CA, USA), which is operated according to the manufacturer’s instructions. Briefly, ESwab containers are vortexed for 5 s, and then 300 µL of the specimen is added to the elution vial, which is vortexed for 10 s. The specimen from the elution vial is then transferred to the assay cartridge. The cartridge is then loaded and run on a GeneXpert instrument. Importantly, each cartridge included a sample processing control (SPC) and a probe check control (PCC). Results of testing are considered invalid if either the SPC or PCC failed. If the result is invalid, the specimen will be retested. The limit of detection (LOD) of the Xpert MRSA NxG assay is between 89 and 812 CFU/mL (9). Importantly, the Xpert MRSA NxG assay only detects the presence of the mecA gene, and therefore, it detects only MRSA.

### Study design

**Fig 1:**
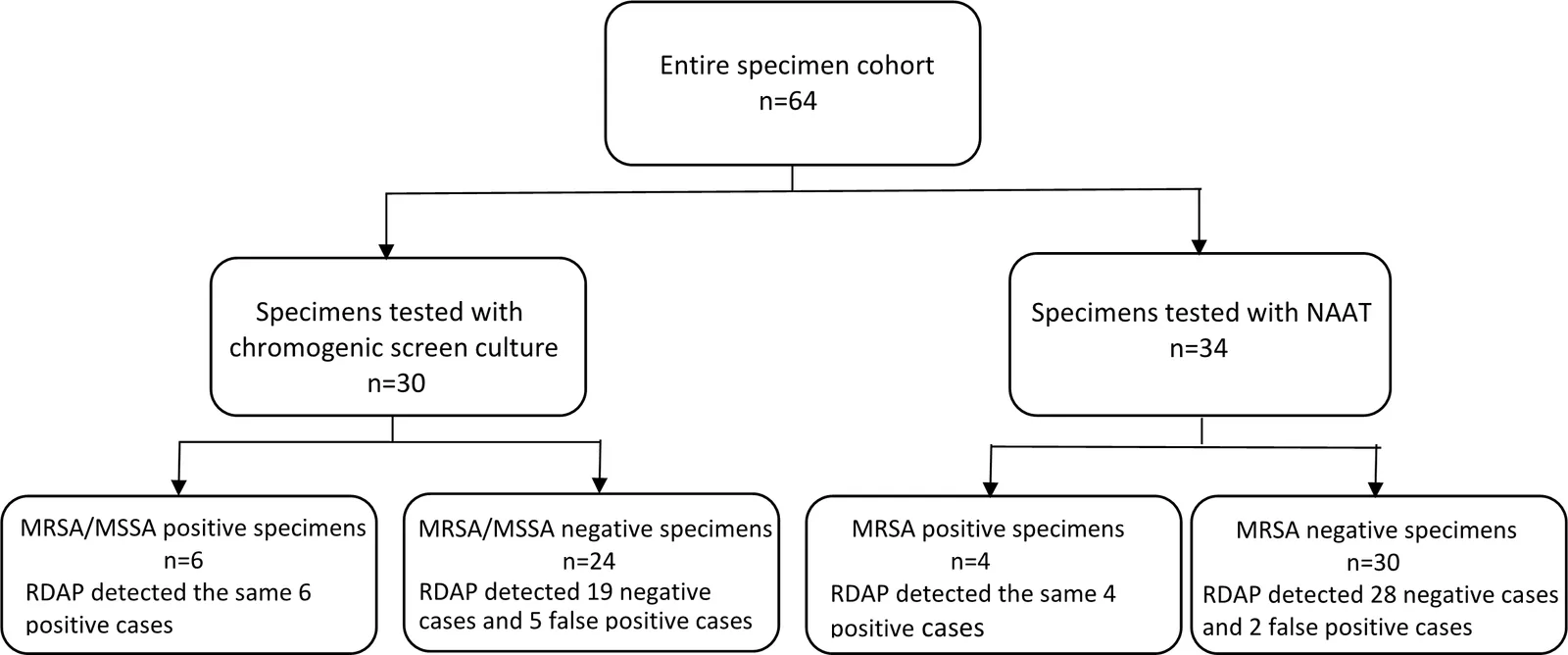
Distribution of clinical specimens between the reference methods, their test results, and the RDAP test results.

Two approaches were used to analyze the specimens. The first approach was for proof of concept. Specimens were first analyzed by the Clinical Microbiology Laboratory (e.g., culture or NAAT), and the remaining portion of the suspensions was stored at 4°C for 24 h until tested with RDAP. The second approach was the blinded design. The specimens were tested by the RDAP either concurrently with the reference method or without the knowledge of the clinical test result. After the lab plated a specimen for culture testing or completed a NAAT test on a specimen, an aliquot of the specimen was made and tested using 30 µL per RDAP channel. For approximately 2 h, the RDAP measurements and the plating process were conducted concurrently. After the RDAP measurements on a specimen were completed, the results were reported to a third party, who released the NAAT result immediately or the culture result >24 h later. Figure 1 shows the distribution of the clinical specimens tested with the reference methods in the blinded tests and the RDAP test results on these specimens.

### Antibodies

The RDAP system uses an antibody-based immunodetection strategy. Commercial antibodies were used to form sandwich immune complexes. For MSSA detection, Anti-Protein A (chicken) Antibody (Rockland Immunochemicals, 200-901-077) was used as the capture antibody (Ab^c^), and Anti-Protein A (goat) Antibody Peroxidase Conjugated (Rockland Immunochemicals, 200-103-077) was used as the detection antibody (Ab^d^). For MRSA detection, Chicken Anti-PBP2a Antibody (RayBiotech, 130-10715) was used as Ab^c^, and peroxidase-conjugated Rabbit Anti-PBP2a (RayBiotech, 130-10073) was used as Ab^d^.

### The RDAP system

RDAP incorporates an electrochemical sandwich immunoassay with field effect electrochemical detection (FEED) (10), which is an ultrasensitive bio-detection technique with voltage-controlled intrinsic signal amplification. Because of its signal amplification capacity, RDAP allows direct detection of bacteria in low-concentration samples without using culture to enrich bacterial concentrations. A detailed description of the principle of RDAP is available in previous publications (10–12). Briefly, a conventional three-electrode electrochemical system is modified with an insulated gating electrode. The redox enzyme horseradish peroxidase (HRP) is conjugated to the Ab^d^ and immobilized on the working electrode (WE) via the Ab^c^-bacterium-Ab^d^ sandwich immune complex. Electron transfer between HRP and the WE of the electrochemical system occurs via the entire complex (11). The gating voltage, *V*_*G*_, applied between the gating electrode and WE induces an electric field at the solution-enzyme-electrode interface to reduce the tunnel barrier for electrons. Therefore, the quantum tunnel current between WE and HRP, which is the signal current, is amplified by *V*_*G*_. The signal amplification allows direct detection of bacteria in extremely low concentrations (single-digit CFU/mL) unprocessed samples, leading to rapid, ultrasensitive, quantitative detection. RDAP features ultra-sensitivity provided by FEED and a high degree of analyte selectivity due to the immunoassay. A detailed description of RDAP is given in the supplemental material.

**Fig 2:**
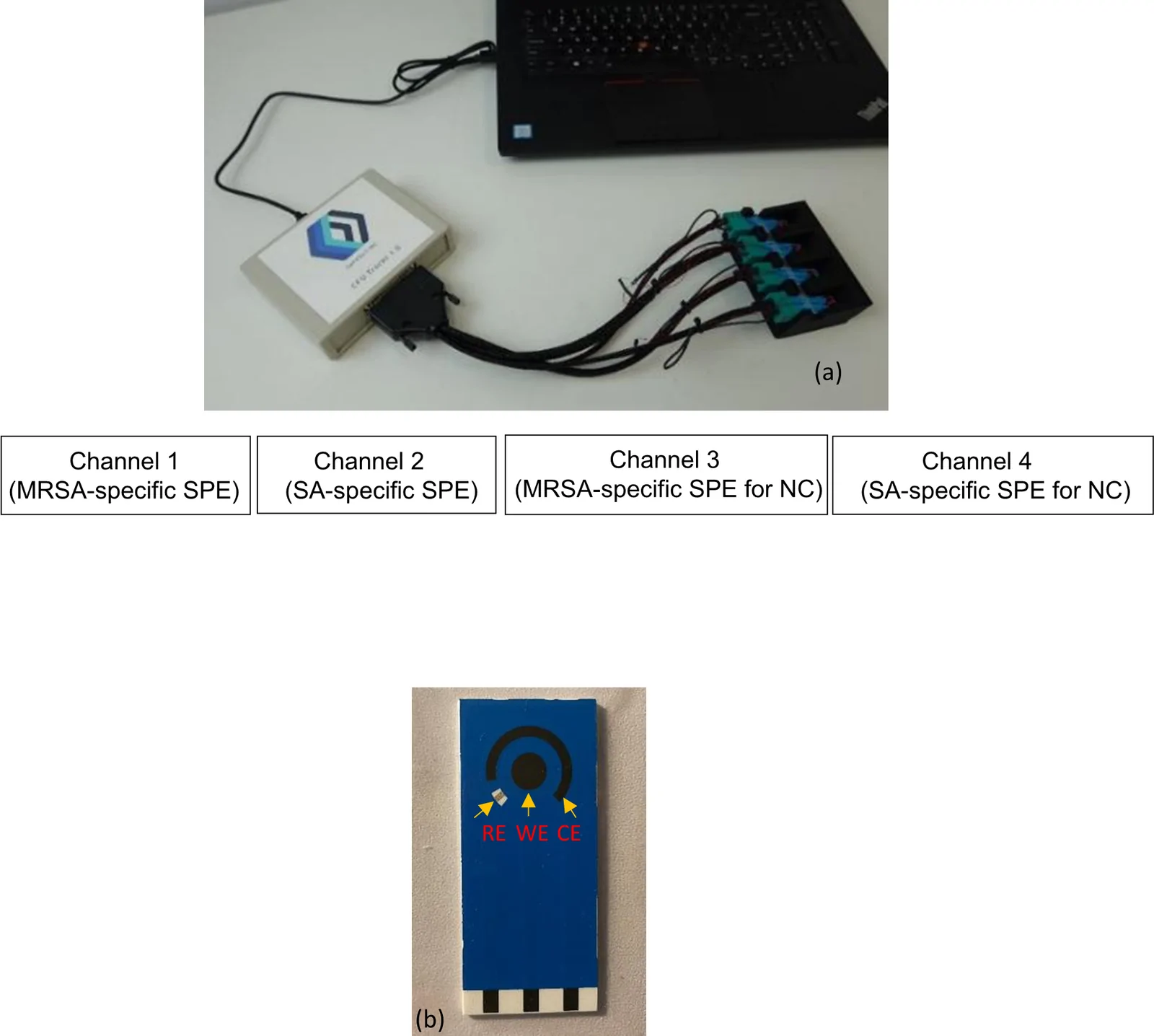
The RDAP prototype. (**a**) The RDAP prototype with multiplexing capability. The prototype consists of a four-channel electronics console, a set of four bacteria-specific SPEs mounted on an electrode holder, and a laptop computer. The four detection channels are connected to four bacteria-specific SPEs used to perform detection and differentiation of MRSA and MSSA and to eliminate detection interference. (**b**) The custom SPE. The carbon WE, silver RE, and carbon CE are fabricated on the top side of the SPE.

The RDAP prototype used to perform the MRSA-MSSA tests consisted of an electronics console, a set of four bacteria-specific screen-printed electrodes (SPEs) mounted on an electrode holder, and a laptop computer as shown in Fig. 2a. The console housed a four-channel potentiostat and a power supply, which was used to apply *V*_*G*_ to the gating electrode assembled on each SPE. The four channels of the potentiostat were connected to the four SPEs, providing multiplexing capability for the prototype to perform simultaneous measurements on the SPEs. Commercial custom SPEs were produced by ZensorR&D (Taichung City, Taiwan). Figure 2b shows the custom SPE. The circular WE had a diameter of 7 mm and was made with graphite powder. The WE, the silver reference electrode (RE), and the graphite counter electrode (CE) were fabricated on the top side of the SPE. The total dimensions of the SPE were 6.1 cm × 2.5 cm × 0.3 cm.

The WE was modified with a composite of carbon nanotube, Nafion, and glutaraldehyde. Briefly, a mixture of single-walled carbon nanotubes (0.2 grams nanotube in 100 mL of 99% dimethylformamide), Nafion (0.5 wt% in ethanol and water), and glutaraldehyde (3% in water) in a volume ratio of 1:1:3 was deposited on the carbon WE until the composite became dry. The modification procedure is described in an earlier publication (13). To construct the detection electrode, Ab^c^ was immobilized on the modified WE via an overnight incubation at 4°C, followed by washing the electrode with de-ionized water (DI) and blocking the non-specific binding sites on the WE with bovine serum albumin. After the Ab^c^ of a bacterium was immobilized on the WE of a SPE, the SPE became a detection SPE specific for that bacterium.

Detection was performed directly on ESwab specimens at room temperature without additional sample processing or culture enrichment. The detection procedure started with depositing a drop of 30 µL of the sample on the modified WE, followed by a 60-min sample incubation at room temperature. The sample was then removed from the WE surface, and the WE was washed with DI. Then, a 30-µL drop of a solution containing Ab^d^ was incubated on the WE for 30 min, followed by washing the WE with DI. At this point, the Ab^c^-bacterium-Ab^d^ sandwich immune complex was formed on the WE, and the SPE was ready for signal detection and analysis. We have conducted an analytical validation of the RDAP following the guidelines for laboratory-developed tests (14) (see supplemental material). The characterized items included linear range, LOD, and precision. Previously, we demonstrated an LOD of 4 CFU/mL (8). The present RDAP prototype demonstrated an LOD of 3 CFU/mL.

### Detection signal analysis

**Fig 3:**
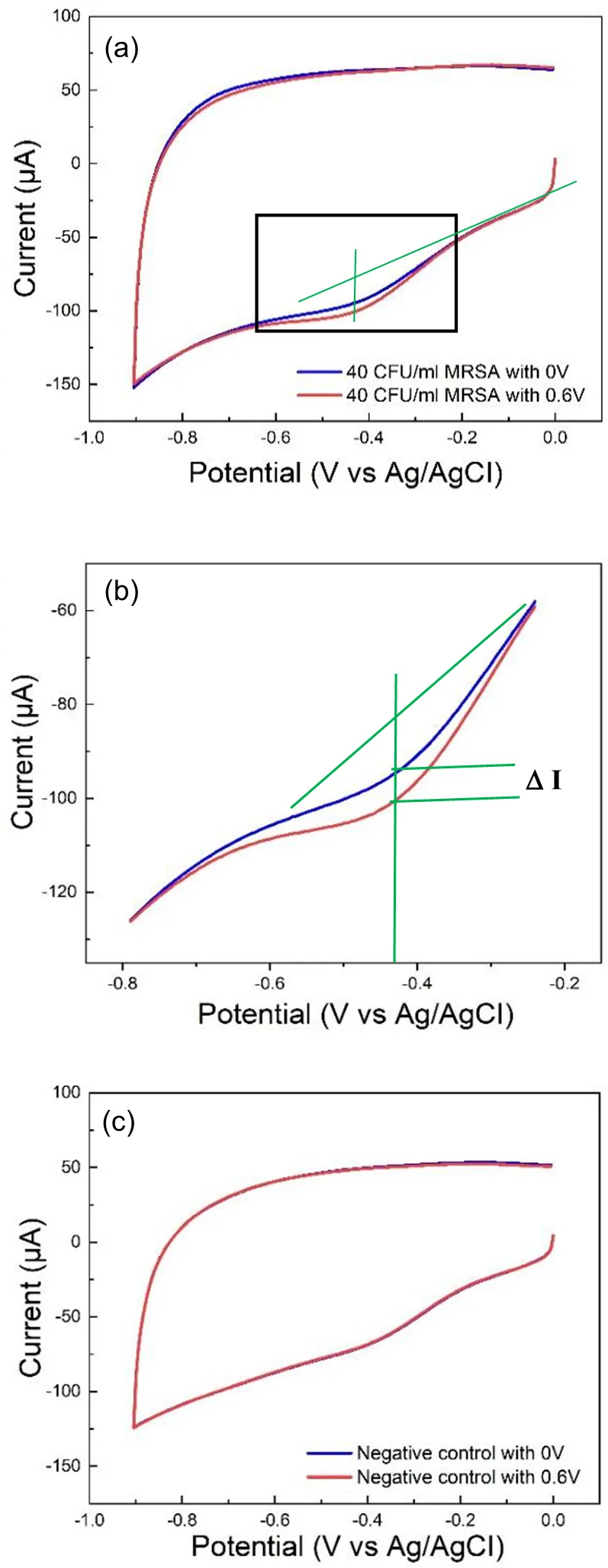
Detection signals of RDAP. (**a**) Amplification of the detection signal from a sample with 40 CFU/mL of MRSA. The reduction peak (−0.44 V) current of HRP measured using the green dashed lines, the slanted line being the baseline line to the CVs, is the detection signal. (**b**) Enlarged view of the reduction peak. The difference between the amplified peak (*V*_*G*_ = 0.6) and the unamplified peak (*V*_*G*_ = 0) is denoted ΔI. (**c**) CVs of a negative control sample with and without *V*_*G*_ amplification.

Detection signals were extracted from the cyclic voltammograms (CVs) of the bacteria-specific SPEs generated by the electrochemical system of the RDAP. Figure 3 illustrates the detection signal analysis using samples prepared with liquid Amies medium. The blue CV in Fig. 3a was obtained with an MRSA-specific SPE that contained an MRSA sandwich complex from a prepared sample without applying *V*_*G*_. The CV shows a peak at −0.44 V, which is the position of the HRP reduction peak. The red CV was obtained with the application of *V*_*G*_ = 0.6 V, which amplified the HRP reduction peak. The difference between the two peaks is denoted by ∆*I* (Fig. 3b) and is used as the indicator of the detection of the target bacteria. The detection signal is the current of the reduction peak of HRP (measured from the baseline) in the red CV (Fig. 3a and b). The signal’s magnitude is proportional to bacterial concentration. The signal can be amplified to a certain extent by increasing *V*_*G*_, leading to *V*_*G*_-controlled amplification of the detection signal. The absence of ∆*I* (i.e., ∆*I* = 0) indicates that the sample does not contain the target bacteria. Figure 3c shows the CVs of a negative control (NC) sample. The amplified HRP reduction peak (i.e*.,* ∆*I* = 0) is not observed due to the absence of the sandwich immune complex on the working electrode. Because the detection mechanism of RDAP is based on the specific immunoreaction between bacteria and their antibodies, the detection of a bacterium also identifies the bacterium in a polymicrobial sample, leading to simultaneous detection and identification. This process is accomplished using a set of bacteria-specific SPEs.

### Statistical analyses

Descriptive statistics, including test sensitivity, specificity, negative predictive value, positive predictive value, and percent agreement, are reported.

## RESULTS

### Discrimination tests

The RDAP’s ability to differentiate between MRSA and MSSA depends on the specificities of the antibodies used for the detection of these strains. Because Protein A is a characteristic surface protein of *S. aureus*, an anti-Protein A antibody was immobilized on the SPE connected to Channel 2 of the RDPA to indiscriminately identify *S. aureus*, which includes MSSA and MRSA, from other microorganisms (15). Protein A, a characteristic surface protein of *S. aureus*, is coded by the *spa* gene. The frequency of this gene in MRSA vs MSSA strains is nearly identical. However, *spa* shows a variation in length in different strains. It was found that the length of the *spa* gene depends either on resistance to methicillin or the source of *S. aureus* isolation (16). In some strains of *S. aureus*, protein A is unable to adhere to the cell wall and therefore is released into the media. This is mainly seen among MRSA strains (16). Protein A has a primary function to bind the Fc domain of IgG (17). These observations may explain the experimentally observed signal appearance of MRSA and MSSA from Channel 1 and Channel 2 described below. Since MRSA is evolved from *S. aureus* (or MSSA), cross-reaction may occur in the detection of these strains. To eliminate the cross-reaction in the detection of MRSA, we used the anti-PBP2a antibody to distinguish between MRSA and MSSA. *S. aureus* develops methicillin resistance through the acquisition of the *mecA* gene, which encodes the low-affinity penicillin-binding protein PBP2a (18). Therefore, this protein, which is specific to MRSA only, is immobilized on the SPE connected to Channel 1 of the RDAP for the exclusive detection of MRSA.

The four detection channels of the RDAP prototype shown in Fig. 2 were used to detect and differentiate MRSA and MSSA and to eliminate detection interference caused by cross-reaction. Channel 1 and Channel 2 were connected to an MRSA-specific SPE and a *S. aureus*-specific SPE, respectively, generating the detecting signals of these microorganisms. Channel 3 and Channel 4 were used to test an NC sample to authenticate the detection signals generated by the signal channels. Liquid Amies medium, the liquid contained in the ESwab, was used as an NC sample. Experimentally, we found MRSA always produced a signal from the MRSA channel and a smaller or sometimes no signal from the SA channel, whereas MSSA always produced a signal from the SA channel and no signal from the MRSA channel. The two NC channels showed no signals. These observations allowed us to distinguish MRSA from MSSA.

**Fig 4:**
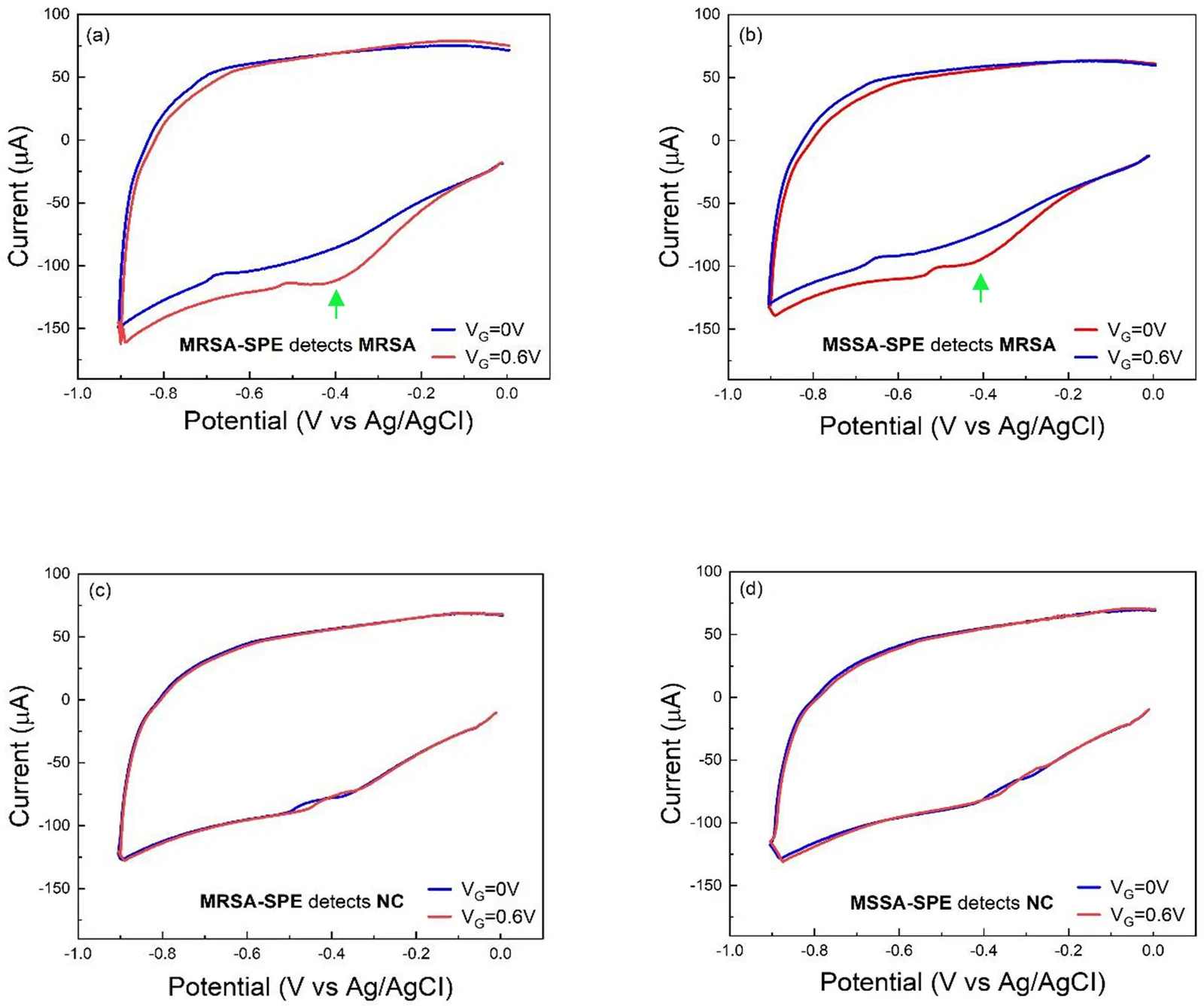
RDAP detection on an MRSA clinical sample. The detection signals were obtained from (**a**) Channel 1 (the MRSA-specific SPE) and (**b**) Channel 2 (the SA-specific SPE). The green arrows in the figures indicate detection events. (**c and d**) The detection results from Channel 3 and Channel 4 on NC samples (Amies medium).

Figure 4 shows the prototype’s detection results on an MRSA clinical ESwab sample (BAC-010), which was confirmed using a chromogenic screening agar test. Figure 4a and b, respectively, shows the signals obtained from Channel 1 (the MRSA-specific SPE) and Channel 2 (the SA-specific SPE). The arrows in the figures indicate positive detection events. The detection of MRSA in Fig. 4a was due to the immune reaction between the PBP2a on MRSA and the anti-PBP2a antibody used to prepare the MRSA-specific SPE. The detection of MRSA in Fig. 4b was due to the reaction between Protein A on MRSA (since MRSA evolved from *S. aureus*) and the anti-Protein A antibody used to prepare the SA-specific SPE. Therefore, when detection signals are generated from both SPEs, the detection interpretation is MRSA. Figure 4c and d show the result of using Channel 3 and Channel 4 to detect an NC sample, which was the Amies medium in the swab container. Figure 4c and d show the absence of the signal, indicating that MRSA or MSSA was not detected. Confirming the lack of signal is essential to authenticate the detection results generated by the other two channels. Taken together, the RDAP prototype correctly detected MRSA.

**Fig 5:**
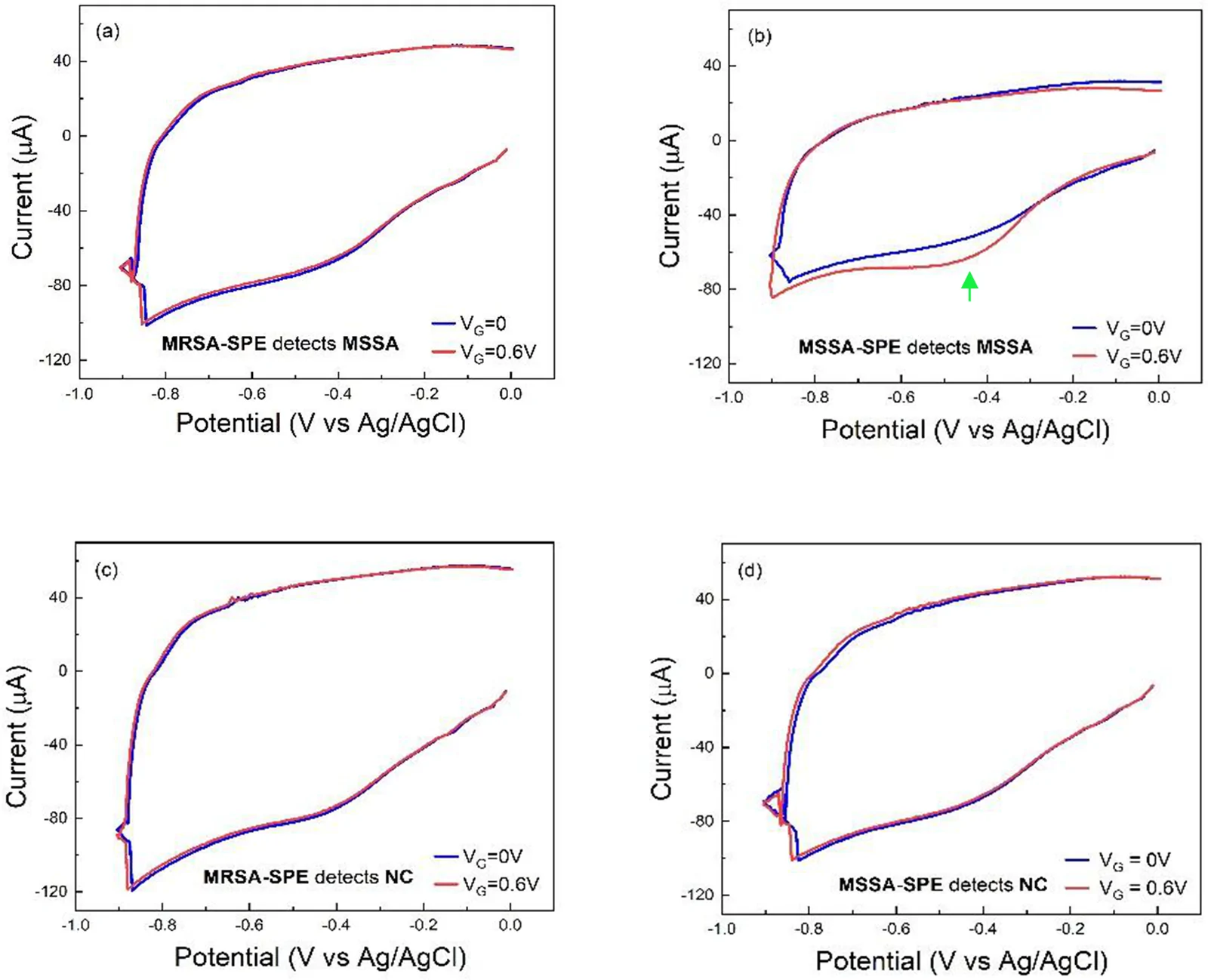
RDAP detection on an MSSA clinical specimen. (**a**) Channel 1 (the MRSA-specific SPE) shows the absence of the detection signal, indicating the absence of MRSA in the specimen. (**b**) Channel 2 (the SA-specific SPE) shows the detection signal of MSSA as indicated by the green arrow. Panels **c and d**, respectively, show the result of using Channel 3 and Channel 4 to detect the NC sample, showing the absence of the detection signal.

Figure 5 shows the prototype’s detection results on an MSSA clinical ESwab sample (BAC-034). Figure 5a shows the absence of the detection signal from Channel 1 (the MRSA-specific SPE), indicating the absence of MRSA in the samples. This conclusion was based on the fact that PBP2a is expressed by MRSA. The arrow in Fig. 5b shows the detection signal of MSSA via the immune reaction between Protein A on MSSA and the anti-Protein A antibody used to prepare the SA-specific SPE. Therefore, the detection results generated by the prototype indicate only the presence of MSSA in the sample. Figure 5c and d, respectively, shows the result of using Channel 3 and Channel 4 to detect the NC sample, showing the absence of the detection signal. Taken together, the RDAP prototype correctly detected MSSA.

### Specificity testing

**Fig 6:**
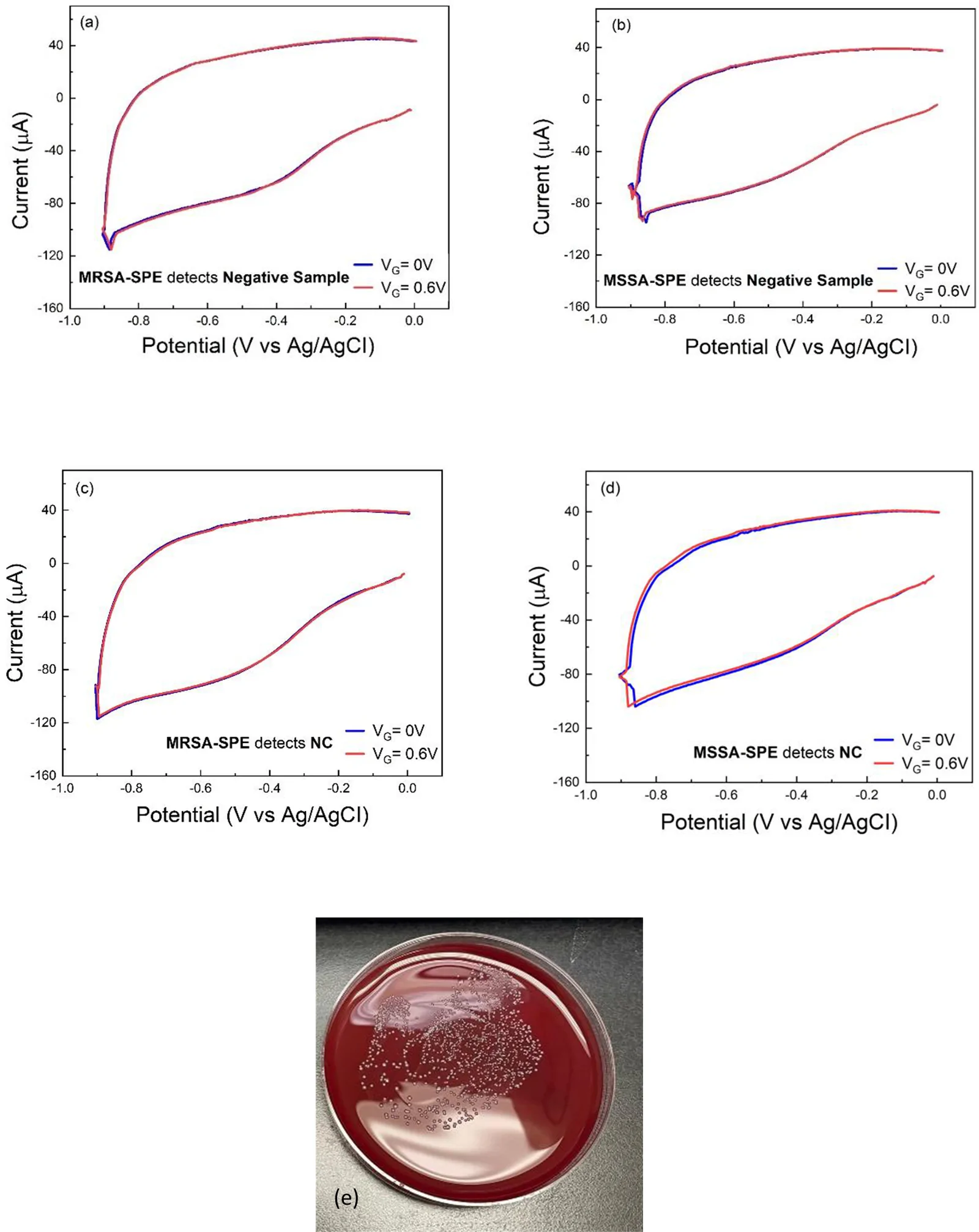
Specificity of RDAP against bacteria other than MRSA and MSSA. Panels **a and b**, respectively, show the absence of MRSA and MSSA in a clinical specimen. Panels **c and d**, respectively, show the absence of the signal from Channel 3 and Channel 4 as expected for NC. Panel **e** shows the result of regular agar plating of this MRSA/MSSA-negative sample, showing bacterial growth caused by skin/mucous flora.

As stated above, an anti-Protein A antibody was used in the assay to indiscriminately identify *S. aureus*, inclusive of MSSA and MRSA. A clinical ESwab specimen (BAC-040) was tested using RDAP to evaluate the specificity of the prototype for *S. aureus*. Routine chromogenic screening agar test on this specimen showed no MRSA/MSSA growth. Figure 6a and b, respectively, shows the results from Channel 1 and Channel 2, showing the absence of a detection signal and, therefore, indicating the absence of MRSA or MSSA in the specimen, thereby confirming the screening agar results. Figure 6c and d shows the absence of the signal from Channel 3 and Channel 4, respectively, as expected for NC. However, Fig. 6e is the result of regular agar plating of this MRSA/MSSA negative sample, showing bacterial growth caused by skin/mucous flora. Figure 6 indicates the specificity of RDAP with respect to mixed skin flora contained in the specimen.

### Blinded testing using culture-based reference

To further evaluate the performance of the RDAP, blinded testing was performed on 30 clinical specimens in parallel with chromogenic screen culture. Clinical swab specimens were collected from patients and then transferred to the clinical microbiology laboratory. After the lab started the plating process, we received a portion of the excess specimen for RDAP tests. Without further processing, 30 mL of the specimen was deposited on each of the two SPEs connected, respectively, to Channel 1 and Channel 2 for RDAP measurements. For approximately 2 h, the RDAP measurements and the plating process were conducted concurrently. After the RDAP measurements were completed, results were returned to the honest broker (a third party), who would later release the culture-based results after ~24 h.

Table 1 shows the detailed information of the blind tests. The information on the comparison testing on clinical specimens is presented according to the test method, i.e., chromogenic screening culture (the reference method) and RDAP. For each specimen, its ID, the TAT achieved, and test outcomes produced using a test method are presented in a sectionalized row of the table. The RDAP section also shows the detection results from its four channels. The specimen ID is also a hyperlink to the plots of the detection CVs from the four channels.

**TABLE 1:** Comparison of RDAP prototype and chromogenic screen culture using clinical specimens^*a*^

Chromogenic screen culture			RDAP					
Specimen ID	TAT	Outcome	MRSA chan	SA chan	MRSA NC chan	SA NC chan	TAT	Outcome
BAC-1296	25 h 1 min	MRSA	Signal	No signal	No signal	No signal	2 h 55 min	MRSA
BAC-1313	24 h 33 min	Negative	No signal	No signal	No signal	No signal	2 h 33 min	Negative
BAC-1321	24 h 48 min	Negative	No signal	No signal	No signal	No signal	2 h 40 min	Negative
BAC-1340	24 h 41 min	Negative	No signal	No signal	No signal	No signal	2 h 37 min	Negative
BAC-1373	27 h 18 min	Negative	No signal	No signal	No signal	No signal	2 h 36 min	Negative
BAC-1383	24 h 31 min	Negative	No signal	No signal	No signal	No signal	2 h 13 min	Negative
BAC-1384	25 h 22 min	Negative	No signal	No signal	No signal	No signal	2 h 30 min	Negative
BAC-1420	26 h 3 min	Negative	No signal	No signal	No signal	No signal	2 h 31 min	Negative
BAC-1424	26 h 30 min	Negative	No signal	No signal	No signal	No signal	2 h 30 min	Negative
BAC-1432	25 h 42 min	Negative	No signal	No signal	No signal	No signal	2 h 20 min	Negative
BAC-1474	29 h 35 min	MRSA	Signal	Signal	No signal	No signal	2 h 12 min	MRSA
BAC-1574	24 h 35 min	Negative	No signal	No signal	No signal	No signal	2 h 9 min	Negative
BAC-1691	18 h 8 min	MSSA	No signal	Signal	No signal	No signal	2 h 15 min	MSSA
BAC-1699	26 h 22 min	Negative	No signal	No signal	No signal	No signal	2 h 30 min	Negative
BAC-1754	33 h 21 min	Negative	No signal	No signal	No signal	No signal	2 h 27 min	Negative
BAC-1755	33 h 15 min	Negative	No signal	No signal	No signal	No signal	2 h 27 min	Negative
BAC-1756	26 h 15 min	Negative	Signal	Signal	No signal	No signal	2 h 15 min	MRSA
BAC-1762	28 h 11 min	Negative	No signal	No signal	No signal	No signal	2 h 50 min	Negative
BAC-1763	26 h 45 min	Negative	Signal	Signal	No signal	No signal	2 h 19 min	MRSA
BAC-1767	25 h 33 min	MSSA	No signal	Signal	No signal	No signal	2 h 12 min	MSSA
BAC-1792	24 h 32 min	Negative	No signal	No signal	No signal	No signal	2 h 25 min	Negative
BAC-1802	27 h 46 min	Negative	Signal	Signal	No signal	No signal	1 h 31 min	MRSA
BAC-1981	25 h 33 min	Negative	No signal	No signal	No signal	No signal	2 h 5 min	Negative
BAC-1982	24 h 47 min	Negative	Signal	No signal	No signal	No signal	2 h 35 min	MRSA
BAC-1986	26 h 1 min	Negative	No signal	No signal	No signal	No signal	2 h 35 min	Negative
BAC-1987	24 h 52 min	Negative	No signal	No signal	No signal	No signal	2 h 24 min	Negative
BAC-1993	33 h 29 min	Negative	No signal	No signal	No signal	No signal	2 h 31 min	Negative
BAC-2023	26 h 15 min	Negative	No signal	Signal	No signal	No signal	2 h 15 min	MSSA
BAC-2408	31 h 34 min	MRSA	Signal	Signal	No signal	No signal	2 h 35 min	MRSA
BAC-2422	34 h 39 min	MRSA	Signal	Signal	No signal	No signal	2 h 48 min	MRSA

^*a*^ The RDAP measurements were performed and completed on the same day as the UH lab started its testing.

The screening culture method detected four MRSA specimens (BAC-1296, BAC-1474, BAC-2408, and BAC-2422) and two MSSA specimens (BAC-1691 and BAC-1767). The remaining 24 specimens tested negative for MRSA and MSSA growth. The RDAP correctly detected all 6 (6/6 or 100%) positive cases and 19 of the 24 (19/24 or 79.2%) negative cases. For the five disagreements (i.e., false positives), four specimens (BAC-1756, BAC-1763, BAC-1802, and BAC-1982) were found to contain MRSA using the RDAP, despite culture having no growth for any *S. aureus*. The last disagreement (BAC-2023) was the RDAP detecting MSSA and culture having no growth for any *S. aureus*. The overall agreement was 25/30 or 83.3%. There were no false-negative results detected by the RDAP prototype.

### Blinded testing using NAAT reference

A challenge of using screening agars as the reference method is that several studies have reported that NAAT offers a superior LOD and has demonstrated similar “false-positive” rates as the aforementioned RDAP results (19). To further investigate the performance of the RDAP against a NAAT method, the Xpert MRSA NxG assay was used as a second reference method in blind testing using 34 new clinical ESwab specimens. Importantly, the NAAT method does not detect MSSA. Table 2 shows the test results. The NAAT method detected four MRSA-positive specimens (BAC-2497, BAC-2498, BAC-2554, and BAC-2558), and RDAP correctly detected all four MRSA-positive specimens (4/4 or 100%). Out of the 30 MRSA-negative specimens tested by NAAT, RDAP found 28 MRSA-negative specimens (28/30 or 93.3%). The two disagreement specimens (BAC-1688 and BAC-2054) had MRSA per RDAP, potentially false-positive results. Of note, regarding two MRSA-negative specimens (BAC-2073 and BAC-2513) tested by NAAT and confirmed by RDAP, RDAP actually identified MSSA in these specimens, as shown in Table 2. Taken together, RDAP had 32/34 (94.1%) overall agreement with NAAT, and no false-negative results were detected by the RDAP prototype.

**TABLE 2:** Comparison of RDAP prototype and NAAT-based testing using clinical specimens^*a*^

NAAT			RDAP					
Specimen ID	TAT	Outcome	MRSA chan	SA chan	MRSA NC chan	SA NC chan	TAT	Outcome
BAC-1562	1 h	Negative	No signal	No signal	No signal	No signal	2 h 5 min	Negative
BAC-1563	1 h	Negative	No signal	No signal	No signal	No signal	2 h 5 min	Negative
BAC-1586	1 h	Negative	No signal	No signal	No signal	No signal	1 h 55 min	Negative
BAC-1588	1 h	Negative	No signal	No signal	No signal	No signal	2 h 10 min	Negative
BAC-1648	1 h	Negative	No signal	No signal	No signal	No signal	2 h 1 min	Negative
BAC-1649	1 h 8 min	Negative	No signal	No signal	No signal	No signal	2 h 10 min	Negative
BAC-1650	1 h 6 min	Negative	No signal	No signal	No signal	No signal	2 h 5 min	Negative
BAC-1661	1 h 2 min	Negative	No signal	No signal	No signal	No signal	2 h 7 min	Negative
BAC-1662	1 h	Negative	No signal	No signal	No signal	No signal	2 h 12 min	Negative
BAC-1688	1 h	Negative	Signal	No signal	No signal	No signal	2 h 12 min	MRSA
BAC-2028	1 h 3 min	Negative	No signal	No signal	No signal	No signal	2 h 30 min	Negative
BAC-2030	1 h 2 min	Negative	No signal	No signal	No signal	No signal	2 h 11 min	Negative
BAC-2039	1 h 5 min	Negative	No signal	No signal	No signal	No signal	2 h 25 min	Negative
BAC-2045	1 h 9 min	Negative	No signal	No signal	No signal	No signal	2 h 20 min	Negative
BAC-2047	1 h 9 min	Negative	No signal	No signal	No signal	No signal	2 h 35 min	Negative
BAC-2054	1 h 9 min	Negative	Signal	Signal	No signal	No signal	2 h 15 min	MRSA
BAC-2063	1 h 9 min	Negative	No signal	No signal	No signal	No signal	2 h 12 min	Negative
BAC-2064	1 h 9 min	Negative	No signal	No signal	No signal	No signal	2 h 41 min	Negative
BAC-2073	1 h 9 min	Negative	No signal	Signal	No signal	No signal	2 h 16 min	MSSA (MRSA negative)
BAC-2074	1 h 9 min	Negative	No signal	No signal	No signal	No signal	2 h 16 min	Negative
BAC-2099	1 h 5 min	Negative	No signal	No signal	No signal	No signal	2 h 11 min	Negative
BAC-2495	1 h 57 min	Negative	No signal	No signal	No signal	No signal	2 h 38 min	Negative
BAC-2497	1 h 41 min	MRSA	Signal	No signal	No signal	No signal	2 h 33 min	MRSA
BAC-2498	2 h 27 min	MRSA	Signal	Signal	No signal	No signal	2 h 29 min	MRSA
BAC-2501	2 h 30 min	Negative	No signal	No signal	No signal	No signal	2 h 39 min	Negative
BAC-2507	1 h 28 min	Negative	Signal	Signal	No signal	No signal	2 h 38 min	MRSA
BAC-2511	1 h 23 min	Negative	No signal	No signal	No signal	No signal	2 h 32 min	Negative
BAC-2513	1 h 27 min	Negative	No signal	Signal	No signal	No signal	2 h 35 min	MSSA (MRSA negative)
BAC-2518	1 h 21 min	Negative	No signal	No signal	No signal	No signal	2 h 37 min	Negative
BAC-2550	1 h 41 min	Negative	No signal	No signal	No signal	No signal	2 h 39 min	Negative
BAC-2554	1 h 22 min	MRSA	Signal	Signal	No signal	No signal	2 h 35 min	MRSA
BAC-2556	1 h 53 min	Negative	Signal	Signal	No signal	No signal	2 h 33 min	MRSA
BAC-2558	1 h 23 min	MRSA	Signal	No signal	No signal	No signal	2 h 34 min	MRSA
BAC-2560	1 h 19 min	Negative	No signal	No signal	No signal	No signal	2 h 39 min	Negative

^*a*^ The RDAP measurements were performed and completed on the same day as the UH lab started its testing.

### Turnaround times

An important component of a diagnostic assay is TAT, which was also evaluated during the above blind testing. Table 1 shows that the average TAT of the 30 specimens tested using the culture-based approach was 27 h 17 min, whereas the average TAT for the RDAP prototype was 2 h 25 min for both comparison studies. The TAT of the 34 specimens tested using NAAT was 1 h 18 min, whereas the average TAT for RDAP was 2 h 21 min (Table 2). The overall average TAT of the RDAP in this study was 2 h 23 min, and all RDAP testing was completed under 3 h.

## DISCUSSION

Screening for MRSA and *S. aureus* (MSSA) plays an important role in infection prevention and control, as well as in potential therapeutic decision-making. For example, empiric treatment regimens may not include MRSA coverage. The presence of MRSA may necessitate escalation of treatment regimens since many empiric coverages do not include MRSA coverage. Another example is that early identification of negative specimens can expedite decision-making for antimicrobial de-escalation. Antimicrobial de-escalation reduces unnecessary broad-spectrum antibiotic use, minimizing the risk of resistance development, adverse effects, and costs. The early identification of negative samples also relieves pharmacy and the lab from the pressure of ensuring safe and effective use of vancomycin.

Screening of MRSA and *S. aureus* is usually performed by NAAT or screening culture. Both approaches have limitations. NAAT testing offers quicker TAT, but reagents are more expensive, and some assays are unable to detect MSSA. On the other hand, culture is typically more cost-effective, but the TATs are typically 16–24 h. There is an opportunity to improve diagnostic capabilities for MRSA and MSSA detection. Here, a new technology, the RDAP, was evaluated for proof-of-concept and preliminary clinical performance.

This early proof-of-concept study revealed that the RDAP can identify MRSA, MSSA, and negative specimens. With the NAAT and screening culture as the test reference methods, using clinical specimens, the blinded study showed that RDAP demonstrated an overall agreement of 89.1% (57/64). All seven discrepancies were from RDAP detecting MRSA or MSSA in specimens tested negative by reference methods. There are two major plausible explanations. First, the RDAP detected false-positive signals, which is unlikely given the antibody specificity and technology. A second explanation is that the reference methods failed to detect MRSA and MSSA and may have an inferior LOD compared to RDAP. This explanation is supported by the demonstrated LOD of the RDAP, which is in the single-digit range, i.e., 3 CFU/mL (see Supplemental material), whereas the LOD of the GeneXpert MRSA NxG assay is 89–812 (22–151) CFU/mL (9), and the average LOD of screen culture under lab conditions is 100 CFU/mL (20). In fact, both explanations are supported by the observed excellent measurement results from the NC specimens, which suggest that the measured detection signals from the culture/NAAT-tested negative specimens are real, indicating the presence of bacteria.

In general, the LOD of NAAT systems is largely determined by the protocol, test methodology efficiency, workflow, and specimen volume being evaluated. For example, the NAAT performed here used one fixed sample size of 300 µL. Thus, under the assumption of 100% efficiency, the calculated LOD for a single viable cell is no lower than 3.3 CFU/mL. In practice, testing is not 100% efficient, and a higher LOD is to be expected. In the GeneXpert MRSA NxG 510K submission, the LODs ranged from 89 to 812 CFU/mL for different isolates (9). As for culture’s LOD, this study inoculated 10 µL to each half of the bi-plate, which calculates to 100 CFU/mL per side. The LOD of the current RDAP prototype was shown to be 3 CFU/mL (see supplemental material). The major advantage of the RDAP is that the targeted analytes are in higher abundance than a single viable cell (agar) or genetic material (NAAT). In light of these considerations, RDAP may offer improved diagnostic capabilities.

The RDAP approach has potential advantages of a short TAT, analyte capacity, low specimen volume, and cost. As mentioned above, the RDAP results were generated with an average TAT of about 2 h 23 min, with no test requiring more than 3 h. This TAT is superior to culture and is more similar to NAAT. Timely results can have important impact on transmission intervention and therapy optimization. Often, the number of target bacteria of a NAAT system is limited to one target due to cost considerations. In the present study, the NAAT system detects only MRSA. On the contrary, RDAP can detect and distinguish both MRSA and MSSA. Table 2 shows that, in two cases (BAC-2073 and BAC-2513), the RDAP identified MSSA in specimens that were reported as negative for MRSA using NAAT. The hyperlinks to the detection results of these specimens show that only the SA channel of RDAP showed the detection signal, which is indicative of the detection of MSSA. In fact, similar RDAP detection results that are indicative of MSSA were also observed in MSSA specimens BAC-1691 and BAC-1767 in Table 1, which were confirmed with culture. It should also be noted that the RDAP can detect bacteria using only 60 µL per run. Finally, the cost of RDAP testing is potentially low because the technology is based on disposable SPEs. We have previously shown that RDAP can differentiate viable from non-viable bacteria by monitoring the growth of viable bacteria (7, 8). Since the RDAP system requires intact bacteria, this may decrease the probability of positive results in the setting of ongoing antibacterial therapy.

While this study offers an early insight into the RDAP technology, it is not without limitations. One limitation is that the specimens used in this study were acquired from a single clinical laboratory, but future work will expand testing to more diverse *S. aureus* isolates. In addition, this study evaluated one reference method per specimen, but future work will compare both reference methods per specimen to better understand the discordance between the RDAP and reference results. Finally, this technology cannot detect MRSA and MSSA from the same specimen. As a note, the current incubation and wash steps of the SPE preparation procedure prior to the RDAP measurements were executed in a serial process for individual SPEs. Future execution of these steps will be based on a parallel process, leading to further reduced TAT. As a final note, because the frequency of the occurrence of positive MRSA/MSSA cultures of the designated lab is 18% and the specimens tested in the studies were not consecutive samples, the RDAP has recorded a low occurrence of positive samples as reflected in Tables 1 and 2. Nevertheless, for both reference methods, the negative predictive value and sensitivity were 100%. Future large-scale clinical studies will be necessary to make more conclusive claims on sensitivity and specificity with statistical certainty and will require a comprehensive testing protocol for discordant results that includes quantitative culture (CFUs/mL) paired with digital droplet PCR and whole-genome sequencing to determine ground truth for discordant results.

### Conclusion

In conclusion, the RDAP technology is a promising new approach for detecting and distinguishing MRSA and MSSA directly from clinical specimens. One of its greatest potentials demonstrated in this study is utilizing its high negative predictive value to rule out *S. aureus* and MRSA, ultimately aiding transmission intervention methods and reducing unnecessary broad-spectrum antimicrobial use, minimizing the risk of resistance development, avoiding potential adverse effects, and costs. It is possible that false-positive results due to high analytic sensitivity of the RDAP platform could lead to unnecessary isolation, antibiotic overuse, and increased healthcare costs. This was a proof-of-concept study and not designed to adjudicate discordant results. Future studies will require a comprehensive testing protocol to evaluate ground truth and clinical relevance.

## Data Availability

The specimen IDs in Tables 1 and 2 are hyperlinks to the RDAP detection results stored in Google Drive.

## SUPPLEMENTAL MATERIAL

Supplemental materials spectrum.00487-26-s0001.docx Note S1 and S2, and Table S1.

ASM does not own the copyrights to Supplemental Material that may be linked to, or accessed through, an article. The authors have granted ASM a non-exclusive, world-wide license to publish the Supplemental Material files. Please contact the corresponding author directly for reuse.
