# Supplementary material for: A rapid approach to detect methicillin-resistant and susceptible Staphylococcus aureus directly from clinical specimens
Source: Microbiol Spectr. 2026 Aug 4;14(9):e00487-26. doi: 10.1128/spectrum.00487-26 (PMC13532265; doi:10.1128/spectrum.00487-26)
Supplement: Supplementary file 1 [file spectrum.00487-26-s0001.docx]

**Supplemental Material**

**A Rapid Approach to Detect Methicillin Resistant and Susceptible Staphylococcus aureus**

**Directly from Clinical Specimens**

Shivani Sharma^1^, Habeeb D. Salaudeen^2^, E. M. Ransom^3^, J. Scott VanEpps^4,5,6^ & Siu-Tung Yau^1,2^

1. Rapidect Inc., Solon, Ohio, USA
2. Department of Electrical Engineering and Computer Science, Cleveland State University, Cleveland, Ohio, USA.
3. The University Hospitals, Cleveland, Ohio, USA; Department of Pathology, Case Western Reserve University School of Medicine, Cleveland, Ohio, USA

4. Department of Emergency Medicine, University of Michigan, Ann Arbor, Michigan, USA.

5. Weil Institute for Critical Care Research and Innovation, University of Michigan, Ann Arbor, Michigan, USA.

6. Biointerfaces Institute, University of Michigan, Ann Arbor, Michigan, USA.

Correspondence author E.M. R. (email: [Eric.Ransom@case.edu](mailto:Eric.Ransom@case.edu)); J.S.V. (email: jvane@med.umich.edu); S.-T.Y. (email: [s.yau@csuohio.edu](mailto:s.yau@csuohio.edu))

V_G_

V_Cell_

Gating Electrode

Gating

Electrode

Working Electrode

Reference Electrode

Counter Electrode

·

·

·

·

·

·

·

·

·

·

·

·

·

·

·

·

·

·

·

·

·

·

·

·

·

·

·

·

·

·

·

·

·

·

·

·

·

·

Bacteria cell

Capture antibody

Detection antibody

Enzyme

Phosphate buffer solution

.

.

.

.

.

.

.

**Figure S1** Schematic description of the RDAP system.

**Supplementary Note 1 - RDAP system**

Field effect enzymatic detection (FEED)^1^ is the basic detection mechanism of RDAP. The FEED system consists of a conventional three-electrode electrochemical cell with a cell potential V_cell_ connected between the working electrode (WE) and the reference electrode.

A redox enzyme used as the sensing element of FEED is immobilized on the WE. The isolation of the enzyme’s active site by a polypeptide network leads to reduced electron transfer between the enzyme and WE, creating a fundamental limit to the sensitivity and therefore the limit of detection for amperometric biosensors^2^. The cell contains an electrolytic solution. An electric double layer is present near the surface of WE. The cell is modified with an insulated gating electrode for applying a gating voltage V_G_ between the gating electrode and the WE, upon which the redox enzyme is immobilized. V_G_ modifies the interfacial charge distribution. When V_G_ is positive, additional negative charges are induced on WE and additional positive ions from the solution are induced at the solution-enzyme-electrode interface. Some positive ions are able to set up electric fields within the polypeptide network in the presence of the transferring electrons residing at the enzyme’s active sites. Quantum tunneling of electrons from the active site through the polypeptide barrier to WE gives rise to the signal current^2^. The energy barrier can be lowered by the electric field so that the tunneling rate is enhanced^3,4^. The result of this process is an amplified signal current, an intrinsic property of the system. Figure S1 shows the components of the FEED system with the exception that the redox enzyme is not directly immobilized on the working electrode.

RDAP incorporates FEED with the immuno-sensing methodology, whose most essential component is the immune complex that is immobilized on the working electrode. Figure S1 shows the capture antibody-bacteria-detection antibody (Ab^c^-bacteria-Ab^d^) sandwich immune complex, in which the enzyme used as the sensing element is conjugated to Ab^d^. The enzyme immobilized on the WE of FEED is replaced by the sandwich immune complex. In addition to the intrinsic signal amplification provided by FEED, RDAP also features a mediator-less operation: the transfer of electrons between the enzyme and WE occurs through the complex without using diffusive mediators^5^.

**Supplementary Note 2 - Analytical validation**

We have conducted an analytical validation of RDAP following the guidelines for laboratory-developed tests^6^. The validation was performed with contrived MRSA samples prepared by mixing MRSA in the Eswab liquid (liquid Amies medium). The linear range was found to be 4-3000 CFU/mL (10 concentrations; 3 replicates on same day).

The LOD of RDAP was empirically determined by measuring contrived MRSA samples at 3 CFU/mL (8 replicates over 5 days) prepared by serial dilutions of a liquid amies medium. The measurement results are shown in Table S1. Each link in Table S1 shows the measurements on a 3 CFU/mL MRSA sample and the measurements on a negative control (NC) sample, which was the liquid amies medium without MRSA. The MRSA measurements all show a signal peak near -0.5 V indicated by an arrow whereas the measurements on the NC samples do not show the signal peak.

Three concentrations, 4, 50, 1000 CFU/mL, that cover the low, intermediate and high concentration ranges were used to indicate the precision of the platform. The 95% confidence interval (95%CI) is a measure of the precision of an assay. At 3 CFU/mL, the platform yields a 95% CI value of 3.58 – 4.20 CFU/mL (based on 3 replicates over 5 days), indicating a high level of precision. Additionally, RDAP has been tested with 20-30 samples of liquid Amies medium (negative control) to ensure its ability to produce true negative results.

Table S1

| [Day 1](https://drive.google.com/file/d/1d9yHfm642mdQ7if9OHJ308mIoW8v-ztw/view?usp=sharing) | [Day 2](https://drive.google.com/file/d/1fgTJtsuw33fbjMuN6EXnoJ5ZugPVDT_Y/view?usp=sharing) | [Day 3](https://drive.google.com/file/d/121MohBAFsrAcNN2xFD5TTCVIQzHhPYBM/view?usp=sharing) | [Day 4](https://drive.google.com/file/d/1YF2CP4Rue4yBgbmDDHFmggQ1PFC1vEI_/view?usp=sharing) | [Day 5](https://drive.google.com/file/d/1LLPcwzuqxMZwpvAkxjoRZsDvQ7asgYYc/view?usp=sharing) |
| --- | --- | --- | --- | --- |

**References**

1.	Choi, Y. & Yau, S.-T. A Field-Effect Enzymatic Amplifying Detector with Pico-Molar Detection Limit. *Analytical Chemistry* **81**, 7123-7126 (2009).

2.	Csoregi, E., Gaspar, S., Niculescu, M., Mattiasson, B. & Schuhmann, W. (eds. Cuyper, M.D. & Bulte, J.W.M.) 105 (Kluwer Academic Publishers, Dordrecht, Boston, London, 2001).

3.	Tans, S. J., Verschueren, A. R. M. & Dekker, C. Room-temperature transistor based on a single carbon nanotube. *Nature* **393**, 49-52 (1998).

4.	Yau, S.-T. & Qian, G. A prototype protein field-effect transistor. *Applied Physics Letters* **86**, 103508 (2005).

5. Wang, J., Xu, Y. & Yau, S.-T. Mediatorless Immunoassay with Voltage-Controlled Intrinsic Amplification for Ultrasensitive and Rapid Detection of Microorganism Pathogens. *ChemElectroChem* **1**, 741 –746 (2014).

6. Burd, E. M. Validation of Laboratory-Developed Molecular Assays for Infectious Diseases. *Clinical Microbiology Rev*iew **23**, 550–576 (2010).
